# Physiological and psychological effects of a 12-week home-based telemonitored training in metabolic syndrome

**DOI:** 10.3389/fcvm.2022.1075361

**Published:** 2023-01-10

**Authors:** Éva Máthéné Köteles, Beatrix Rafael, Andrea Korom, Anna Vágvölgyi, Judit Erzsébet Ábrahám, Andrea Domján, Mónika Szűcs, Attila Nemes, Mária Barnai, Csaba Lengyel, István Kósa

**Affiliations:** ^1^Department of Physiotherapy, Faculty of Health Sciences and Social Studies, University of Szeged, Szeged, Hungary; ^2^Department of Medical Prevention, Albert Szent-Györgyi Medical School, University of Szeged, Szeged, Hungary; ^3^Department of Medicine, Albert Szent-Györgyi Medical School, University of Szeged, Szeged, Hungary; ^4^Department of Medical Physics and Informatics, Albert Szent-Györgyi Medical School, University of Szeged, Szeged, Hungary

**Keywords:** telerehabilitation, telemonitoring, metabolic syndrome, home-based, exercise training, psychological factors

## Abstract

**Background:**

Metabolic Syndrome (MetS) increases the risk of cardiovascular diseases (CVD) and affects around one fourth of the population worldwide. In the prevention and treatment regular exercise trainings are inevitable. Providing personal supervision in out/inpatient care settings for such a large target population challenges the healthcare systems, but using telemonitoring of the home-performed trainings could be a promising and widely available option.

**Objectives:**

The aim of this study was to evaluate the physiological and psychological effects of a 12-week home-based physical training program, telemonitored by widely available fitness devices on parameters of MetS patients.

**Methods:**

A total of 55 MetS patients (mean age 49.19 ± 7.93 years) were involved in the study. They were asked to perform 3–5 sessions of exercise activity (min. 150 min) each week for 12 weeks. Trainings were monitored off-line by heart rate sensors, a fitness application and a cloud-based data transfer system. Physiotherapists supervised, coached, and feedback the trainings through an online coach system. We investigated different anthropometric parameters, maximum exercise and functional capacity levels, laboratory parameters, the level of depression, insomnia, vital exhaustion, and wellbeing as well.

**Results:**

The average weekly training time was 152.0 ± 116.2 min. Out of the 55 participants who completed the program, 22 patients (40%) performed the recommended 150 min or more weekly. Patients showed statistically significant changes in: all the measured waist and hip circumferences; 6-min walk distance (6MWD; from 539.69 ± 78.62 to 569.72 ± 79.96 m, *p* < 0.001); maximal exercise capacity (11.02 ± 2.6 to 12.14 ± 2 MET, *p* < 0.001), stress-electrocardiogram duration time (13.74 ± 3.29 to 15.66 ± 2.64 min, *p* < 0.001); body weight (98.72 ± 21.7 to 97.45 ± 21.76 kg, *p* = 0.004); high-density lipoprotein cholesterol (*n* = 45, 1.28 ± 0.31 to 1.68 ± 0.36 mmol/L, *p* < 0.001); fasting plasma glucose (FPG; *n* = 47, 6.16 ± 1.26 to 5.44 ± 1.31 mmol/L, *p* = 0.001); glycated hemoglobin A1c (HbA1c; *n* = 41, 6.22 ± 0.68 to 5.87 ± 0.78%, *p* = 0.01). Out of the 55 patients who finished the program 38 patients (70%) completed all the psychological questionnaires. We found statistically significant decrease of the overall scores of the Maastricht Vital Exhaustion Questionnaire, from 3.37 ± 2.97 points to 2.63 ± 2.70 points (p < 0.05) and a significant increase of the overall scores of the WHO Wellbeing Scale from 9.92 ± 2.59 points to 10.61 ± 2.76 points (*p* < 0.05). We have not found any statistically significant changes in the scores of the Beck Depression Inventory and the Athens Insomnia Scale.

**Conclusion:**

A 12-week home-based telemonitored training supported by an affordable, commonly available device system produces positive, statistically significant changes in many core components in MetS patients. Telemonitoring is a cheap method for coaching and feeding back the home-based interventions.

## Introduction

Metabolic Syndrome (MetS) is defined by the concomitant presence of cardiometabolic risk factors such as central (abdominal) obesity, high blood pressure, elevated fasting plasma glucose (FPG) and triglyceride levels, and decreased high-density lipoprotein cholesterol level (HDL-C) (1). This condition increases 2–5 times the risk of developing cardiovascular diseases (CVD) and type 2 diabetes mellitus (T2DM) in the next 5–10 years (1) and the occurrence of cardiovascular death by 1.8 times in the next 15 years (2). In developed countries MetS affects around one fourth (20–30%) of the adult population. This ratio is increasing especially amongst the elderly (23.5–40.6%) (3, 4). According to the guideline of the International Diabetes Federation lifestyle interventions, primarily regular physical activity and calorie-restricted healthy diet are essential in other to lose bodyweight and to treat the MetS (5).

Review of the evidence shows that MetS is comorbid with different psychological disorders; obesity (central adiposity) and diabetes (insulin resistance) increase the risk of depression and anxiety. Furthermore depression, anxiety, and MetS all have environmental and behavioral factors in common, increasing their risk of prevalence, such as unhealthy diet, smoking, chronic stress, and physical inactivity. On the other hand after successful weight loss programs the improvement of depression was reported (6, 7). It is also supported by evidence that vital exhaustion (excessive fatigue, loss of energy and demoralization) is an independent risk factor of CVD (8). Based on the findings of two meta-analyses, the relative risks for cardiovascular events are between 1.50 and 2.03 (9, 10).

Changing of the lifestyle is a very cumbersome process; only a very small proportion of the population is able to cope with this alone. The majority of patients require strong coaching, which challenges the Public Health Services, depleting of structural, financial, and human resources. There is another, predetermined conflict pertaining to the patient: a great proportion of the affected patients are in the active age group, where by their occupational activities prohibit the regular visits to outpatient facilities offering therapeutic physical training programs. A further barrier is the time consumption and cost of regular commuting to and from the nearest outpatient clinic. Since the outbreak of the COVID-19 pandemic we should also consider the potential infection hazards related to institutional group training. Services provided through telemedical technology are preferred in this situation to minimize interpersonal contacts between health care workers and patients as well as between patients.

Formal or informal telemedical services are used regularly by a considerable portion of patients. They are measuring their blood sugar and blood pressure in their home regularly and the data are used from a distance by the physician to adjust their drug therapy. Doctors can even diagnose an acute heart attack from a distance through telemedical electrocardiogram systems. Recent studies proved that online telemonitored trainings are as effective as hospital or center based programs in patients with cardiac abnormalities requiring cardiac rehabilitation. These telemonitored programs were characterized by higher patient satisfaction, improved adherence, and higher cost-effectiveness than inpatient or outpatient solutions (11–13).

Few studies have analyzed the psychological effects of home-based telemonitored training in cardiac rehabilitation and most of them only measured changes in quality of life. The results on the quality of life of telehealth intervention are mixed. Some studies have found an improvement in quality of life (14, 15), while other studies have found no significant difference in the increase in quality of life of those treated remotely (16, 17).

The usage of telemedicine for training monitoring of patients with cardiometabolic risk factors but not dependent on close medical supervision could be also a forward-looking decision. This could not only reduce the burden of healthcare facilities, but also save time and cost for the patients. Lots of lifestyle coaching apps are available on the market which make it possible to monitor the actual lifestyle—dietary habits and physical activities—of the patients from a distance. However these solutions are not integrated into the health care system, these are typically practiced only in the field of fitness.

Through the development of infocommunication technologies, “smart” devices are becoming more reliable while with decreasing cost these devices are becoming more broadly available for the general public in everyday life. This advanced technology makes it possible to supervise the home-based training of patients, depicting their heart rate during the training and also delivering information about daylong physical activity. Data are transferred through mobile units to a cloud based datastore, which provides web service for the overview of different aggregation of the home measured data.

## Purposes

The primary purpose of our study was to evaluate the physiological and psychological effects of a 12-week home-based physical training program telemonitored by widely available fitness devices in MetS patients.

## Materials and methods

### Study design and patient recruitment

This study was a prospective non-randomized intervention evaluation study among MetS patients, who were involved in the study between 1st of September 2018 and 31st of January 2020. They were recruited from the city of Szeged (Hungary) and surrounding villages within a 40 km distance. The recruitment was performed in General Practitioners’ offices, occupational physicians’ offices as well as in cardiological inpatients and outpatient facilities. Participants were recruited through a physician’s referral. All subjects were informed about the study protocol and they provided a written informed consent before the enrollment in the study. The study was done in accordance with the Declaration of Helsinki and the study protocol was approved by the Hungarian Medical Research Council (ETT TUKEB), the Ethical Trial Number is 50780-2/2017EKU. Our Clinical trial registration number is NCT05146076.

### Inclusion criteria

Voluntary patients, aged between 25 and 70 years, were involved in the study, who practiced only low level of regular physical activity (self-reported, less than 30 min a week), and had at least three risk factors concomitantly from the followings (1):

(a) waist circumference above 102 cm in men and above 88 cm in women,
(b) proved type 2 diabetes mellitus or FPG level above 5.6 mmol/L,
(c) treated hypertension or spontaneous blood pressure ≥ 130/85 mmHg,
(d) treated hypertriglyceridemia (HTG) or serum triglyceride (TG) level above 1.7 mmol/L,
(e) serum HDL-C level under 1.03 mmol/L in men, under 1.3 mmol/L in women

In order to manage the telemonitoring devices and data transfer the participants had to be able to use a smartphone and to transfer data from a smart watch to a phone, laptop or personal computer through a wired or wireless (Bluetooth) connection.

### Exclusion criteria

Participants were excluded with: any upcoming planned invasive cardiological intervention (percutaneous transluminal coronary angioplasty, coronary artery bypass, valve repair or replacement), uncontrolled hypertension (blood pressure > 160/100 mmHg), type 1 diabetes mellitus (T1DM), T2DM which needed more than one dose of insulin per day, chronic heart failure, chronic renal failure where the estimated Glomerular Filtration Rate < 60 ml/min, oncological diseases, serious cognitive disfunction, lack of cooperation, any known disease or condition that seriously affected the mental and legal capacity, any other conditions preventing regular physical trainings.

### Primary and secondary outcome measures

When planning our study, we set two primary outcome measures. One of them was the waist circumference (WC) as it is a basic anthropometric measure to assess abdominal obesity, and its increased proportion serves as one of the risk factors in the MetS by definition (1). Our second primary outcome measure was the 6-min walk distance, the patient’s best achievable walked distance in 6 min performed during the 6-min walk test (6MWT), which is a simple field test commonly used to assess functional capacity (18, 19). We considered all other measured parameters (a series of anthropometric, fitness, body composition, metabolic parameters: the hip circumference, the body weight, the body mass index, the stress-ECG duration time, laboratory parameters such as FPG, glycated hemoglobin, triglyceride, high-density lipoprotein and total cholesterol levels, and quality of life) as secondary outcome measures.

### Baseline and follow-up patient assessment

The baseline and follow-up medical assessments and the data collection took place in the Department of Medicine, Albert Szent-Györgyi Medical School, University of Szeged, (Szeged, Hungary), while the physiotherapy assessments and the relevant data collection were performed in the Department of Physiotherapy, Faculty of Health Sciences and Social Studies, University of Szeged (Szeged, Hungary). Baseline assessments were performed maximum 1 week before starting the training program, while follow-up evaluation was performed within 1 week of completing of the 12-week training program.

#### Anthropometric measurements

The following anthropometric measurements were taken by physiotherapists: Body weight measured by the body composition analyzer device (Tanita BC-418, Japan); height in centimeters using a metric staniometer attached to a wall (20); waist circumference measured in cm at navel level and the narrowest part of the midriff; and hip circumference at the level of the greater trochanter (21). Additional details about the procedures of the measurements are presented in Supplementary material.

#### Functional capacity

The functional capacity was measured during the 6MWT by the physiotherapists, in accordance with the American Thoracic Society guideline, on a 30 m long marked track. The distance reached in 6 min (in meters) was measured and documented (18, 19).

#### Maximal exercise capacity

Under a cardiologist’s supervision a 12-channel electrocardiogram (CardioSys, MDE Diagnostic, Walldorf, Germany) was performed at rest and during exercise using an incremental loading in accordance with the Modified Bruce Protocol until the age-predicted maximal heart rate, where this target heart rate was calculated based on the 220-age formula. The maximal capacity in Metabolic Equivalent of Task (MET; ml/kg/min), the maximal heart rate (bpm) systolic and diastolic blood pressure (mmHg) and the time spent on the maximal exercise level in seconds (s) were measured and documented.

#### Body composition analysis

The body composition analysis was performed with a Bioelectrical Impedance Analysis using a segmental body composition analyzer device (Tanita BC-418, Japan) measuring the following parameters: bodyweight (kg), the total body fat mass (BFM) in kilograms (kg), its ratio referred to the body weight (BFM%), the muscle mass (MM) and, the fat-free mass (FFM) in kilograms (kg). The visceral fat level (VF) in the abdomen and the trunk fat percentage (TF%) were measured with an abdominal fat analyzer device (Tanita ViScan AB-140, Japan). The Body Mass Index (BMI; kg/m^2^) and the average basal metabolic rate (BMR) in Joule (J) were calculated by the device and were documented. Additional details about the procedures of the measurements are presented in Supplementary material.

#### Medical evaluation and laboratory parameters

Anamnesis, detailed medical history, available laboratory data, current drug treatment were reviewed by the physicians and documented at the time of the initial medical evaluation. Based on these data and the assessment of the physical status of the patients, the inclusion, and exclusion criteria were checked. Routine echocardiographic evaluation was also performed using a Vivid-e (Boston, Massachusetts, US) cardiac ultrasound machine. Blood samples from 1 month prior to the starting date were accepted and documented at the initial and the final visit.

#### Psychological questionnaires

During the initial and the final visits the participants were asked to fill in (self-report) the following standardized psychological questionnaires:

1. Shortened Beck Depression Inventory: this self-report instrument contains 9 items from the original 21-item version of BDI, and it is highly correlated with the total score (*r* = 0.92, *p* < 0.001). It is used to determine the intensity and severity of depression. 0–9 points: normal score; 10–18 points: mild depressive mood; 19–25 points: moderate depressive mood; over 25 points: severe depressive mood (22, 23).

2. Athens Insomnia Scale: the scale consists of eight items which measure sleep complaints. The first five pertains to night-time problems, and the other three items assess the negative consequences of disturbed sleep during the day. Respondents are required to rate positively if they have experienced sleep difficulties at least three times per week during the last month. Each item is rated on a 4-point numerical rating scale (where 0 = no problem at all and 3 = very serious problem). Total scores range from 0 to 24. Higher scores in these AIS measures indicate that responders have severe insomnia symptoms (24).

3. Shortened Maastricht Vital Exhaustion Questionnaire: This is the nine-item form of the original 21-item questionnaire which was used to measure feelings of exhaustion (25). This shortened version was applied in a Hungarian representative health survey, Hungarostudy 1995 (26). Higher scores indicate that responders have severe vital exhaustion symptoms. The correlation between the shortened Hungarian version and the original Dutch 21-item scale is high *r* = 0.94, *p* < 0.001 (27).

4. WHO Wellbeing Scale: It is the most common measure which is used to assess self-reported wellbeing in clinical or follow-up studies. Validation of this questionnaire is based on data from Hungarostudy 2002. The Hungarian version of this scale is a reliable and valid measure of positive quality of life. The Cronbach alpha rate of the scale is 0.84, which refers to a high internal consistency. It is a five-item questionnaire which assesses wellbeing during a period of 2 weeks. The raw score ranges from 0 to 25. A score of 0 represents the worst quality of life and 25 the best (28).

#### Intervention: Home-based telemonitored physical trainings

After all the assessments were completed, the physiotherapists informed the participants about the details and features of the trainings and the monitoring. They were given an information sheet and each participant received a 15–30 min individual patient education on the usage of the given training monitor device and the procedures of the data transfer. The participants were asked to perform 3–5 trainings (minimum 30 min per session) individually at home, aiming to a minimum of 150 min per week for 12 weeks. There were no restrictions regarding what type of trainings they performed at home (walking, running, cycling, swimming etc.) but they were informed about the recommended and beneficial training types and intensity suitable for MetS patients. The target heart rate zone for the home based training was calculated individually, based on the exercise capacity of the patients and the perceived level of exhaustion in the 60–80% range of the maximal predicted heart rate. Patients were asked to keep their heart rate within this target zone during the physical activities they performed at home.

#### Training monitoring

The home-based trainings were monitored by two different types of device—a chest strap (Polar H10, Kempele, Finland) wirelessly connected with an android smartphone (Meizu M5c, China) and a free to download fitness application (Polar Beat) or an optical heart rate sensor (Polar M430 GPS running watch, Kempele, Finland)—that were allocated to the patients at the initial visit (29, 30). Both were available in the commercial market for a reasonable price. Additional details about the measuring devices and the procedures of the measurements are presented in Supplementary material. The patients were exercising at home on at least 3 days of the week. The physiotherapists reviewed the training activity of every patient weekly and contacted them individually via phone or email giving feedback and trying to motivate the participants. Average time expenditure for an individual consultation was about 10–20 min. Supplementary Figure 1 shows a diary view of the trainings performed by a participant, while Supplementary Figure 2 indicates some features of a monitored training that were visible on the website for the monitoring person.

#### Statistical methods

Power analysis for the study was performed using the software G* Power (Version 3.1.9.2) for power-and-sample size calculation (University of Düsseldorf, Germany). The calculated sample size was 51 based on waist circumferences, working with an effect size *d* = 0.45, alpha as Type I error of 0.05, and a power value of 0.9. Statistical data were reported as the mean ± standard deviation (SD). Paired *t*-test was used to analyze the effect of the training on several parameters, whereas we used correlation analysis to measure the strength of connection between two continuous variables. Statistical tests were performed using R statistical software (R version 3.6.2) (31). Values of *p* < 0.05 were considered significant. Diagrams were made also using R statistical software.

## Results

### Subject population

Altogether 59 MetS patients (37 men and 22 women, mean age 49.35 ± 8.51 years) were enrolled in the study. Four participants (two men and two women) dropped out from the program, although they had started and had been allocated a heart rate monitor device but did not finish the program and refused to take part in the final medical and physiotherapy assessments. The dropout rate was 6.8%. Finally 55 patients (35 men and 20 women, mean age 49.19 ± 7.93 years) completed the 12 week home-based telemonitored program. Table 1 shows the demographic characteristics of the participants. Out of those patients who completed the program, in 4 cases the final exercise electrocardiogram test and in 1 case the final 6MWT could not be performed either because of health related issues (acute knee injury and pain that limited the walking and cycling ability) or technical problems.

**TABLE 1:** Demographic characteristic of the participants.

		Number of patients
Gender	Male	35
	Female	20
Qualification	Primary school	4
	Vocational training	4
	High school	29
	University/Collage	18
Economic activity	Homemaker	1
	Employed	19
	Public servant	14
	Self employed	14
	Unemployed	1
	Retired	6
Family status	Single	4
	Civil partnership	8
	Married	36
	Divorced	7

The average weekly heart rate monitored training time was 152 ± 116.2 min. Out of the 55 participants who completed the program, 22 patients (40%) performed the recommended 150 min or more activity time during a week.

None of the assessors (physicians and physiotherapists) reported any adverse events during the initial and final assessments and none of the patients reported any adverse events during the 12 week home-based training program.

### Changes in waist circumference

**FIGURE 1:**
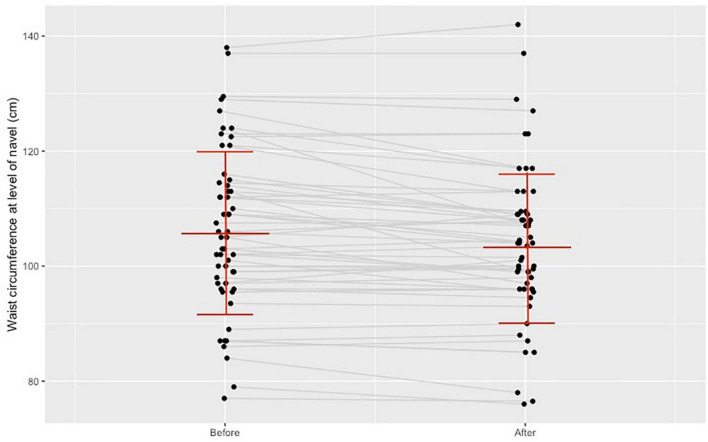
Change in the waist circumference measured at navel level before and after the training program.

Post intervention the average waist circumference measured at the narrowest part of the midriff (WC_midriff_, 106.17 ± 14 to 103.88 ± 13.5 cm, *p* < 0.001) and the average waist circumference measured at navel level (WC_navel_, 112.8 ± 14.8 to 110.6 ± 15.5 cm, *p* = 0.001) significantly reduced as indicated in Figure 1.

Other anthropometric parameters, such as the average hip circumference (HC, 114.73 ± 13.75 cm to 112.15 ± 13.2 cm, *p* < 0.001), the average body weight and the BMI that we considered secondary outcomes also showed statistically significant improvements. Table 2 shows the measured anthropometric parameters at baseline and after the training program.

**TABLE 2:** Anthropometric parameters at baseline and after the training program.

	*n*	At baseline (mean ± SD)	After 12-week training (mean ± SD)	Diff (95% CI)
WC_navel_ (cm)	55	112.82 ± 14.82	110.61 ± 15.53	−2.21 (−3.47; −0.95)**
WC_midriff_ (cm)	55	106.17 ± 14.03	103.88 ± 13.5	−2.29 (−3.40; −1.18)**
HC (cm)	55	114.73 ± 13.75	112.15 ± 13.2	−2.58 (−3.50; −1.66)**
Body height (cm)	55	172.9 ± 9.29	n.a.	n.a.
Body weight (kg)	55	98.72 ± 21.7	97.45 ± 21.76	−1.27 (−2.12; −0.41)*
BMI (kg/m^2^)	55	32.98 ± 6.69	32.58 ± 6.73	−0.41 (−0.68; −0.13)*

SD, standard deviation of the mean; WC_navel_, waist circumference measured at navel level; WC_midriff_, waist circumference measured at the narrowest part of the midriff; HC, hip circumference; BMI, body mass index; n.a., not applicable. Paired *t*-test was used. Level of significance: *p < 0.01, **p < 0.001.

### Changes in functional capacity

**FIGURE 2:**
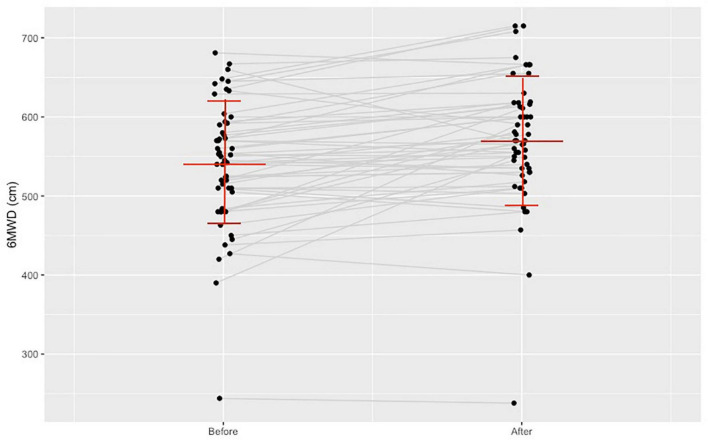
Change in the 6-min walk distance before and after the training program.

As can be seen in Figure 2 the average 6 min walk distance increased (*n* = 54, 6MWD, 539.69 ± 78.62 to 569.72 ± 79.96 m, *p* < 0.001**).** A positive correlation was found between the average weekly training time and the increase of 6MWD (*r* = 0.3; *p* = 0.029).

### Changes observed in the secondary outcome measures

The maximal exercise capacity (*n* = 51, from 11.02 ± 2.6 to 12.14 ± 2 MET, *p* < 0.001), and the time to the maximal exercise level (13.74 ± 3.29 to 15.66 ± 2.64 min, *p* < 0.001) also improved significantly.

### Body composition parameters

Post intervention the body composition parameters had not changed significantly. The overall average body fat mass (BFM) showed a decreasing tendency from 33.22 ± 13.9 to 32.61 ± 14.46 kg (*p* = 0.087), but the average body fat mass relative to the body weight had not changed significantly. Surprisingly, the average fat-free mass (FFM) also showed a decreasing tendency. The average BMR calculated by the device had decreased from 8239.56 ± 1694.38 to 8150.02 ± 1653.45 J (*p* = 0.037). We managed to measure the visceral fat (VF) level in the abdomen and the trunk fat percentage (TF%) in 47 participants, in the case of 8 participants, their increased waist circumference limited their ability to fit under the measuring device. Post intervention the VF level did not change significantly, although the average TF% had shown a declining trend, but the change did not reach significant level. Table 3 shows the measured body composition parameters at baseline and after the training program.

**TABLE 3:** Body composition parameters at baseline and after the training program.

	*n*	At baseline (mean ± SD)	After 12-week training (mean ± SD)	Diff (95% CI)
BFM (kg)	55	33.22 ± 13.9	32.61 ± 14.46	−0.61 (−1.31; 0.09)
BFM (%)	55	33.01 ± 8.26	32.74 ± 8.73	−0.27 (−0.87; 0.33)
FFM (kg)	55	65.51 ± 13.17	64.97 ± 12.99	−0.54 (1.28; 0.19)
MM (kg)		62.4 ± 12.6	61.8 ± 12.5	−0.64 (−1.27; −0.004)*
BMR (J)	55	8239.56 ± 1694.38	8150.02 ± 1653.45	−89.55 (−173.53; −5.56)*
VF	47	18.1 ± 5.89	18.23 ± 6.59	0.14 (−0.68; 0.96)
TF (%)	47	41.4 ± 6.67	41.15 ± 7.51	−0.25 (−1.29; 0.79)

SD, standard deviation of the mean; BFM, body fat mass; FFM, fat free mass; MM, muscle mass; BMR, basal metabolic rate; VF, visceral fat; TF, trunk fat. Paired *t*-test was used. Level of significance: * < 0.05.

### Laboratory parameters

**FIGURE 3:**
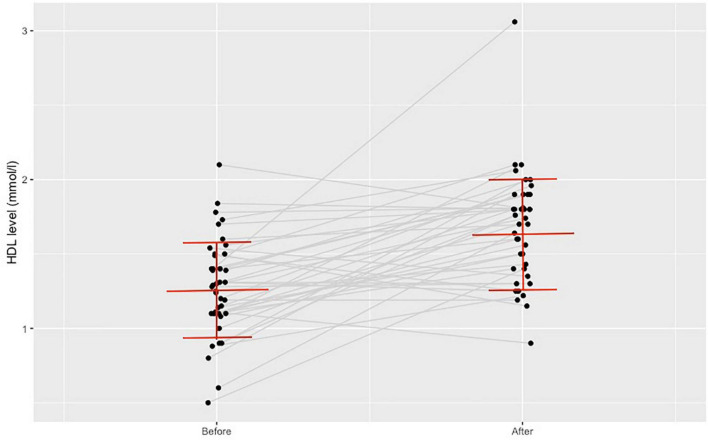
Change in high-density lipoprotein-cholesterol level before and after the training program.

**FIGURE 4:**
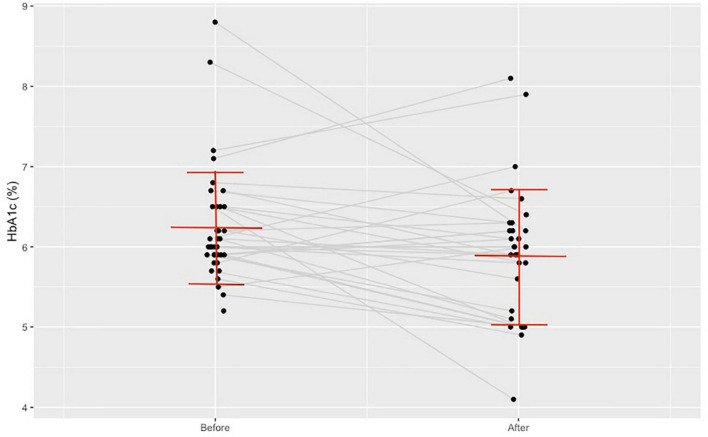
Change in glycated hemoglobin A1c level before and after the training program.

Post intervention the average HDL-C level increased (*n* = 45, from 1.28 ± 0.31 to 1.68 ± 0.36 mmol/L, *p* < 0.001, as indicated in Figure 3) and the average FPG level decreased (*n* = 47, from 6.16 ± 1.26 to 5.44 ± 1.31 mmol/L, *p* = 0,001). In 41 patients we managed to document the glycated hemoglobin (HbA1c) level and it decreased from 6.22 ± 0.68 to 5.87 ± 0.78% (*p* = 0.01), as indicated in Figure 4. There was a declining trend in the triglyceride (TG) level (*n* = 47), but not significantly. The total cholesterol (TC) level did not change. Table 4 shows the documented laboratory parameters at baseline and after the training program. A weak correlation tendency was found between the average weekly training time and the HDL-cholesterol level increase (*r* = 0.23; *p* = 0.137).

**TABLE 4:** Laboratory parameters at baseline and after the training program.

	*n*	At baseline (mean ± SD)	After 12-week training (mean ± SD)	Diff (95% CI)
HDL-C (mmol/L)	45	1.28 ± 0.31	1.68 ± 0.36	0.40 (−0.27; −0.53)**
FPG (mmol/L)	47	6.16 ± 1.26	5.44 ± 1.31	−0.72 (−1.11; −0.32)**
HbA1c (%)	41	6.22 ± 0.68	5.87 ± 0.78	−0.35 (−0.61; −0.09)*
TG (mmol/L)	47	2.19 ± 1.45	1.89 ± 0.55	−0.29 (−0.73; 0.14)
TC (mmol/L)	47	5.66 ± 1.38	5.83 ± 0.94	0.18 (−0.14; 0.49)

SD, standard deviation of the mean; HDL-C, high-density lipoprotein-cholesterol; FPG, fasting plasma glucose; HbA1c, glycated hemoglobin A1c; TG, triglyceride; TC, total cholesterol. Paired *t*-test was used. Level of significance: *p < 0.01, **p < 0.001.

### Psychological questionnaires

Out of the 55 patients who finished the monitored training program we managed to collect the completed, self-reported questionnaires from 38 patients (roughly 70%). These 38 cases were included in the statistics. The overall scores of the Vital Exhaustion Scale decreased (*n* = 38, from 3.37 ± 2.97 points to 2.63 ± 2.70 points, *p* < 0.05) and the overall scores of the WHO Wellbeing Scale increased (*n* = 38, from 9.92 ± 2.59 points to 10.61 ± 2.76 points, *p* < 0.05). The scores of the Beck Depression Inventory and the Athens Insomnia Scale did not change significantly. Table 5 shows the scores of the four psychological questionnaires used at baseline and after the training program.

**TABLE 5:** Psychological Questionnaire’s scores at baseline and after training program.

	*n*	At baseline (mean ± SD)	After 12-week training (mean ± SD)	Diff (95% CI)
BDI (points)	38	2.32 ± 2.78	2.18 ± 3.52	−0.13 (−0.84; 0.58)
AIS (points)	38	2.47 ± 3.20	2.61 ± 4.07	0.13 (−0.54; 0.81)
MQ (points)	38	3.37 ± 2.97	2.63 ± 2.70	−0.74 (−1.44; −0.03)*
WHO-WBS (points)	38	9.92 ± 2.59	10.61 ± 2.76	0.68 (0.04; 1.32)*

SD, standard deviation of the mean; BDI, Beck Depression Inventory; AIS, Athens Insomnia Scale; MQ, Maastricht Vital Exhaustion Questionnaire; WHO-WBS, WHO-Wellbeing Scale. Paired *t*-test was used. Level of significance: * < 0.05.

## Discussion

A key component of the MetS is abdominal or central obesity that can be measured by the increase of waist circumference (1). In our study we have observed a significant decrease in the average WC, a minimal but statistically significant weight loss and BMI decrease over the 12 weeks period. Decrease in the body weight and lowering the waist circumference positively affects abdominal obesity and are proved to decrease other risk factors of MetS like elevated plasma glucose level and hypertension as well (32). Nowadays regular physical exercise is recommended for all diabetic patients, but the management of patients on intensive conservative insulin therapy is relatively complex (33). To avoid this complication in the current, developing phase of telerehabilitation we considered intensive insulin therapy as an exclusion criteria. With the maturation of monitoring tools, in subsequent studies we should also include such patients, because they form an important subgroup of the MetS population.

Our telemonitored MetS patients achieved significant increase in their exercise tolerance and functional capacity. The average maximal exercise capacity increased by 1.12 MET and the stress ECG duration time by roughly 2 min. The 6MWD improved by an average of 30 m. This is according to Bohannon and Crouch within the range of the minimal clinically important difference for change in the 6MWD for adults with pathology (34). It had been previously proved by numerous studies on the MetS that increasing the cardiorespiratory fitness level of these patients plays a key role in decreasing the risk factors and the prevalence of the disease (35). Moreover this will positively affect the workability of these patients. Haufe and colleagues in their recent study involving 314 MetS patients concluded that regular and telemonitoring-supported physical activity decreased MetS severity and increased workability, and this effect was independent of sex and occupation (36). Although their telemonitored intervention took 6 months, they reported bigger weight loss and exercise capacity improvement than in our study, but they could not detect significant HDL-C increase.

Because in the present study patients with MetS were undergoing intervention, we evaluated separately our data based on the cut-off values of individual MetS risk factors. Characteristics of the population regarding the presence of pharmacologically treated diseases (T2DM/Hypertension/Hyperlipidemia), as well as the proportion of abnormal individual MetS risk factors, are listed in Table 6. Drug treatment was unchanged during the intervention, so only changes of continuous variables were listed.

**TABLE 6:** Metabolic syndrome risk factors at baseline and after training program.

	Number of cases at baseline	Number of cases after 12-week training (Percentage of baseline numbers)	
		Improved	Worsened
Treated T2DM	10		
Treated hypertension	37		
Treated hyperlipidemia	16		
Abnormal FPG	35	23 (66%)	3 (9%)
Abnormal blood pressure	48	9 (19%)	1 (2%)
Abnormal TG	28	5 (18%)	10 (36%)
Abnormal HDL-C	12	12 (100%)	1 (8%)
Abnormal WC	50	7 (14%)	2 (4%)
Minimum 3 risk factors	55	6 (11%)	0 (0%)

T2DM, type 2 diabetes mellitus; FPG, fasting plasma glucose; TG, triglyceride; HDL-C, high-density lipoprotein cholesterol; WC, waist circumference.

The average weekly training time reached by our patients meets the physical activity guidelines according to which a minimum of 150 min of moderate-intensity or 75 min of vigorous-intensity physical activity per week is recommended to achieve medical benefits (37, 38). Forty percent of the participants achieved the recommended 150 min or more activity time during a week. This ratio indicates that in the case of the majority of our patients (60%) more efforts should have been applied to the issue of self-motivation.

Promotion of exercise, maintaining interest in physical trainings and enhancing self-motivation long-term are key tasks. With the help of online coach systems the type, the duration, the intensity and other parameters of physical activities performed at home could be continuously followed from a distance by the supervising physiotherapists. Using a fitness device available to the public for telemonitoring, with online feedback from a professional, can lead to a better adherence to the training programs, but the feedback should be regular and motivating. All efforts should be taken to try to increase the activity time. The effect of telemonitoring in the setting of cardiac rehabilitation has been evaluated in several studies. We can find meta-analysis which states that in home-based telemonitored rehabilitation environment the adherence level of participant is higher than in the center-based environment (39). Other meta-analysis was not able to document this relationship (40).

In our study we have found parameters of the body composition showing improving tendencies (total body fat mass alone and relative to the body weight, trunk fat percentage) but have not found any statistically significant changes. One possible explanation for this could be that body composition measurements do not reproduce as well as anthropometric or bodyweight measurements. They can be affected by many factors, therefore detecting significant changes is more difficult. Visceral fat (between the abdominal organs) deposits are associated with systemic inflammation and insulin resistance that play a key role in the development of the MetS. According to evidence of previous studies summarized in a recent review, it is more important to observe reductions in the amount of visceral fat than to see reductions in the body weight or the BMI (41), which we did in our study.

One of our most important findings was that after a 12 week home-based telemonitored training we could observe a significant increase in the HDL-C level of our MetS participants. According to evidence from previous large prospective studies, including the Framingham Heart Study (42), every 1 mg/dL or 0.026 mmol/L increase in the HDL-C level is associated with a 2–3% decrease in the risk of developing CVD. Referring this finding to our recent study, and calculating with a 0.4 mmol/L HDL cholesterol level increase, this could mean an approximately 30% decrease of cardiovascular risks. This improvement, according to some studies, is even better than the improvements that were achieved by using statins or fibrate therapy (43–47). In addition to effectively reducing LDL-C (low-density lipoprotein cholesterol) levels, statins are also able to increase HDL-C level by 6–14.7%. (43–47). The hallmarks of fibrate therapy are a substantial decrease of plasma TG levels ranging from 30 to 50% and a moderate increase of HDL-C levels ranging from 5 to 15% (48). Kodama et al. in 2007 conducted a meta-analysis on 25 studies assessing the effects of exercise training on HDL-C level. They found a modest but significant HDL-C level increase (0.065 mmol/L) as a result of regular aerobic exercise. The minimal amount of exercise volume at which HDL-C level increase occurred was 120 min per week (49). In our study we have obtained bigger HDL-C increase (0.4 mmol/L) with higher weekly training time (152.0 ± 116.2 min).

The FPG level of our MetS patients decreased significantly after the intervention. High level of FPG is by evidence, strongly associated with the risk of developing T2DM. According to the Diabetes Prevention Program (DPP) results 50% of the participants in the lifestyle intervention group had achieved the targeted weight loss (7%) or more by the end of the curriculum (24th week) and 38% had a weight loss of at least 7% at the time of the most recent visit. With an average follow up of 2.8 years, their results showed that lifestyle intervention reduced the incidence of T2DM by 58% compared with placebo (50). Our study was not as effective as the DPP study in regards of body weight reduction as we put the emphasis on the training activity increase, only a few minutes of dietary education were applied during the initial visit.

Regarding the changes in the patients’ psychological state we have to emphasize that the level of vital exhaustion—one of the most important indicators of chronic stress—decreased significantly after the home-based physical training program. Pedersen and colleagues in their cohort study investigated the connection between vital exhaustion and MetS, including 3,621 participants who did not have MetS at the baseline of their study. During the 10 years of follow-up, 186 women (9%) and 120 men (8%) developed MetS and they found that vital exhaustion was associated with a higher risk of MetS, most strongly in men (51).

We found a significant increase in the level of wellbeing as well. Boylan and Ryff’s (52) study examined the association between wellbeing or their components and MetS in 1205 participants in their survey of the Midlife in the US (MIDUS). At their 9–10 years of follow-up, they found a 36.6% MetS prevalence; and life satisfaction, positive affect and personal growth predicted fewer MetS components. They concluded that several dimensions of wellbeing predicted lower risk of MetS (52).

The level of depression and the level of insomnia did not change after the physical training program. It is important to stress that these values were considered normal prior to our telemonitored program.

The latest WHO guideline (53) for physical activity and sedentary behavior recommends physical activity specified for people living with selective chronic conditions (such as T2DM or hypertension). They have found strong evidence that physical activity is associated with decreased risk of CVD mortality and decreased levels of HbA1c, blood pressure, BMI and lipids amongst adults with T2DM. For adults with hypertension, there is high-certainty evidence that physical activity decreases risk of progression of cardiovascular disease and reduces blood pressure, while there is moderate-certainty evidence that physical activity reduces the risk of CVD mortality (53). According to the 2021 European Society of Cardiology Guidelines on cardiovascular disease prevention in clinical practice, consumer-based wearable activity monitors/trackers and home-based and telehealth (mHealth) interventions are now recommended for cardiovascular patients to increase patient participation and long-term adherence to exercise training programs (54).

The long lasting effect of life style intervention is crucial. Project type intervention typically documents favorable short term results, but the prompt withdrawal of telecoaching may abolish the effects. However, decline will never be so prompt as in the case of drug treatment withdrawal. To moderate withdrawal effects more and more studies use patient-owned devices for the intervention, which can be used in the framework of self-care, also after the completion of the telecoached intervention phase. Intermittent late follow-up of the patient can promote the long term effects of the intervention, allowing detection of patient adherence failures, where repeated coaching phases could be helpful. The assessment of late follow-up episodes could be automated, where algorithms evaluate the data and processed the feedback for patients, sparing costly human effort.

If we apply the special consideration of behavior economy by supplying the patients with the monitoring devices, we have the opportunity to utilize special incentives for late adherence to a life style change (55). It was earlier demonstrated that the effectiveness of life style intervention can be improved by appropriate financial contracting of the patients, where the patients undertake some financial burden at the initiation of the intervention with the promise of reimbursement after successful completion of the program. The appropriate fine tuning of such incentive requires detailed studies.

### Limitations

1. We used two different devices that were available on the commercial market for a reasonable price, for monitoring the heart rate during the physical trainings. We intended to gain monitoring experience with the two different types. The comparison of the effectiveness and applicability of the different types of training monitors require further analyses, which exceeds the limits of this publication.

2. In our study we only monitored the home-based physical exercise. Dietary counseling and food intake monitoring were not part of our program, although besides regular physical trainings a healthy diet is the other key element in the complex lifestyle interventions of the MetS. In a running study we will assess the combined effects of exercise and diet.

3. Our home-based telemonitored program lasted for 12 weeks, which is a relatively short period of time. To see results and changes long-term, we should increase the duration of the training program and perform the late follow-up of our patients.

4. Our study ran partially during the 2 years of COVID-19 pandemic. In this time we were limited in gathering some necessary data like the psychological questionnaires from some of our patients.

### Clinical implication

Telehealth facilities, including the telemonitoring of certain human parameters or complete exercise trainings with professional support, will extend the possibility for rehabilitation/lifestyle intervention activities beyond in-clinical service centers or inpatient frames to great a number of MetS patients with distant geographical locations. It could also provide a chance for those patients with time restrictions for participating in a regular daytime outpatient service. Since the outbreak of the COVID-19 pandemic we all experienced an increased demand for these kinds of telemedical solutions to provide a service without much human interaction but with healthcare professionals’ distant supervision. Our program worked, and could work in pandemic situations providing an alternative service in a home-based environment for the increasing number of MetS patients.

## Conclusion

A 12-week home-based telemonitored training supported by an affordable, widely available device system produces positive, statistically significant changes in many core components of the MetS in patients suffering from MetS. Telemonitoring is a cheap method for coaching and providing feedback on home-based interventions. This intervention approach is particularly useful for overloaded healthcare services and during pandemic times, but also has potential for use more broadly to improve the efficiency of healthcare delivery.

## Data availability statement

The datasets presented in this article are not readily available due to local restrictions. Requests to access the datasets should be directed to corresponding author.

## Ethics statement

The study was done in accordance with the Declaration of Helsinki and the study protocol was approved by the Hungarian Medical Research Council (ETT TUKEB), the Ethical Trial Number is 50780-2/2017EKU.

## Author contributions

ÉM, MB, AD, CL, AN, BR, and IK given substantial contributions to the conception, the methodology, and the design of the manuscript. JÁ given substantial contribution in the patient recruitment, selection, and data collection. ÉM, AV, MB, AK, BR, and IK taken part in the investigation, data collection, and the interpretation of the results. MS did the statistical analysis. AD contributed to the project supervision. ÉM participated in writing and drafting the manuscript. MB, AN, BR, and IK revised the manuscript critically. All authors read and approved the final version of the manuscript.
