# Supplementary material for: Physiological and psychological effects of a 12-week home-based telemonitored training in metabolic syndrome
Source: Front Cardiovasc Med. 2023 Jan 10;9:1075361. doi: 10.3389/fcvm.2022.1075361 (PMC9871627; doi:10.3389/fcvm.2022.1075361)
Supplement: Supplementary file 1 [file Data_Sheet_1.docx]

Supplementary material

*Baseline and follow-up patient assessments*

- Anthropometric measurements

Exact body weight was measured by the body composition analyser device (Tanita BC-418, Japan), standing on its scale part, without shoes and heavy clothing. Height was measured in centimeters (cm) using a metric stadiometer attached to a wall. The patients stood erect without shoes, with the back of the head, the shoulder blades, the buttocks and the heels touching the wall and the patient looking directly forward [20]. All circumferences were measured in centimeters (cm) using a stretch‐resistant tape that provides a constant 100 g tension. Hip circumference (HC) was measured at the level of the greater trochanter (around the widest portion of the buttocks), with the tape parallel to the floor. Waist circumference was measured at two levels: at navel level and at the narrowest part of the midriff (at the midpoint between the lower margin of the last palpable rib and the top of the iliac crest). For each measurements, the subjects stood erect, with feet close together, arms at the sides and body weight evenly distributed across the two feet. The subjects were relaxed, and the measurements were taken at the end of the normal expiration phase of the breathing [21].

- Body composition analysis

The body composition analysis was performed with a Bioelectrical Impedance Analysis using a segmental body composition analyser device (Tanita BC-418, Japan). The device measures body composition by measuring bioelectrical impedance in the body using eight electrodes and constant current source with a high frequency current (50kHz, 500μA). The degree of difficulty with which electricity passes through a substance is known as the electrical resistance. Electricity passes through water rather easily but fat within the body allows almost no electricity to pass through. The percentage of fat and other body constituents can be inferred from the measurements of this resistance. Electric current is supplied from the electrodes on the tips of the toes of both feet and the fingertips of both hands, and voltage is measured on the heel of both feet and the thenar side of both hands. The current flows into the upper limbs or lower limbs, depending on the body part(s) to be measured, five different impedance measurements to be made – whole body, right leg, left leg, right arm and left arm. During the measurement the exact bodyweight (kg), the total body fat mass (BFM) in kilograms (kg), its ratio referred to the body weight (BFM%), the muscle mass (MM) in kilograms (kg), the fat free mass (FFM) in kilograms (kg) were detected and analysed. The visceral fat level (VF) in the abdomen and the trunk fat percentage (TF%) were measured withby an abdominal fat analyser device (Tanita ViScan AB-140, Japan). The Body Mass Index (BMI; kg/m2) and the average basal metabolic rate (BMR) in Joule (J) were calculated by the device and were documented.

- Training monitoring

The home-based trainings were monitored by two different types of device - a chest strap (Polar H10, Kempele, Finland) wirelessly connected with an android smartphone (Meizu M5c, China) and a free to download fitness application (Polar Beat) or an optical heart rate sensor (Polar M430 GPS running watch (Kempele, Finland) - that were allocated to the patients at the initial visit [25, 26]. Both were available in the commercial market for reasonable price. The chest strap (Polar H10, Kempele, Finland) has an electric heart rate sensor. For patients using this type of device data collection was initiated and stopped through the free to download fitness application (Polar Beat) on the paired smartphone. Training duration, distance, intensity and heart rate zones could be reviewed by the patient on the smartphone online as well as after completing the training. At the end of the training the data were also synchronized automatically to a cloud based integrated system for reviewing and coaching [25, 26]. The smart watch (Polar M430 GPS running watch, Kempele, Finland) has an optical heart rate sensor that is capable of starting and ending the trainings by itself, but the synchronization of training data is not automatic. Patients using this type of sensor had to upload the training data by manually building up a wired connection to a PC running the Polar Flow website, or through initiating a wireless connection to a patient own smartphone running the Polar Flow application by pressing a buttom on the smartwatch. The browser based review process was identical for the two groups. The assignment of devices to individual patients was planned on a random manner, but during the inclusion procedure we should realise, that a considerable part of the patients disprefered the sensor system containing chest strap. Accepting these dispreferences to ensure the inclusion of effective patient number, patients using smart watches were relatively overrepresented.

Supplementary Figures


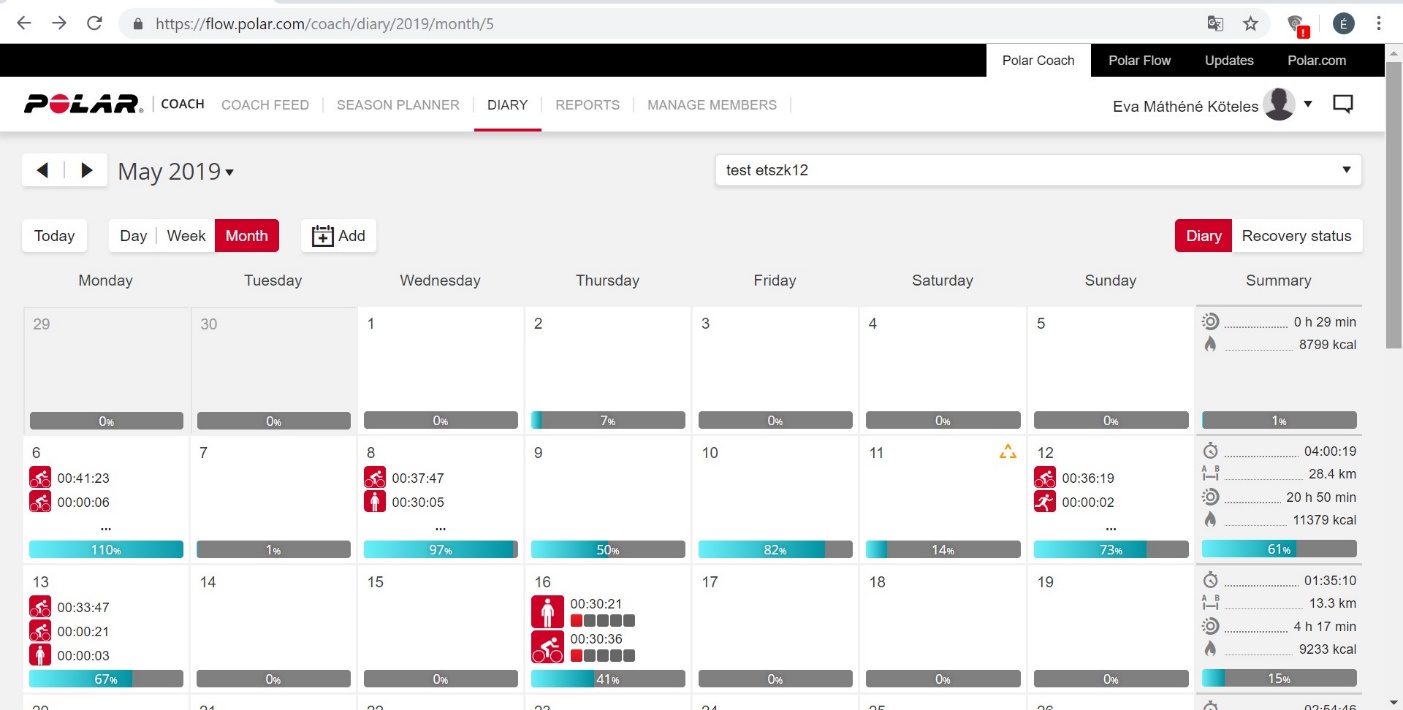


Supplementary Figure 1. -Printscreen picture of the weekly performed trainings from the Polar Flow coaching website


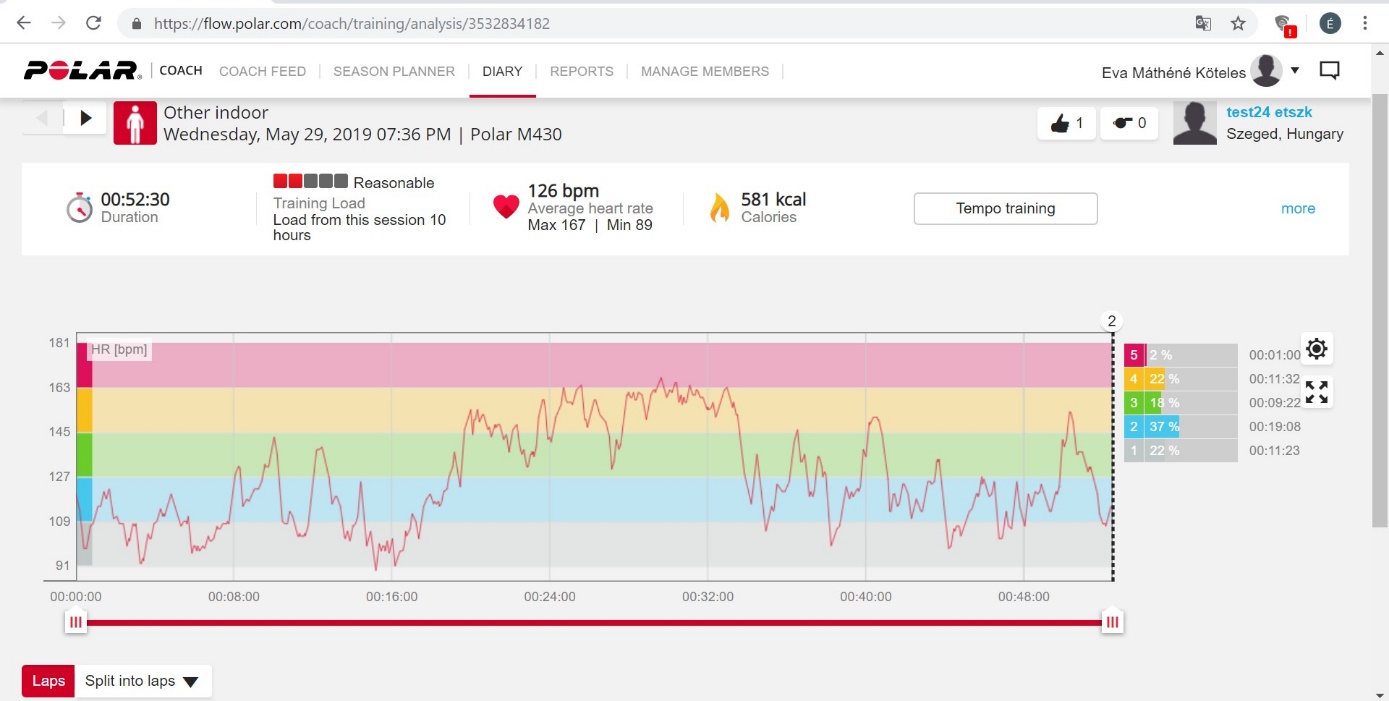


Supplementary Figure 2. - Printscreen picture of a monitored training session from the Polar Flow coaching website
